# BPSD reconsidered: diagnostic considerations to preserve personhood in persons with dementia

**DOI:** 10.3389/frdem.2023.1272400

**Published:** 2023-11-22

**Authors:** Alison Warren

**Affiliations:** The Department of Clinical Research and Leadership, George Washington University School of Medicine and Health Sciences, Washington, DC, United States

**Keywords:** BPSD, NPS, dementia, Alzheimer's disease, stigma, personhood

## Abstract

BPSD is relatively common but profoundly disturbing to persons with dementia, their family, and caregivers. Growing recognition of the impact of BPSD on quality of life has improved recently, but assessment and management approaches are still lacking. Considerable controversy surrounding the label of BPSD has garnered a great deal of attention, with implications of its contribution to the already pervasive dementia-related stigma experienced by persons with dementia and their caregivers. This brief review aims to summarize salient viewpoints, controversies, and considerations of the assessment, management, and perception of BPSD, in an effort to offer potential recharacterizations of BPSD to promote and prioritize personhood in persons with dementia.

## Introduction

Dementia has rapidly become a major global health problem and can result from various diseases (Kwon and Lee, 2021), with Alzheimer's disease (AD) as the most representative and common form, affecting over 50 million people worldwide (Alzheimer's disease facts figures, 2023). While the most salient feature of AD is progressive memory impairment, behavioral and psychological symptoms of dementia (BPSD) may also emerge over the progression of the disease process with considerable variability and heterogeneity (Swierkosz-Lenart et al., 2019). BPSD is an umbrella term that characterizes neuropsychiatric (i.e., non-cognitive) manifestations including, but not limited to, agitation; irritability; depression; anxiety; delusions; (Swierkosz-Lenart et al., 2019; Felstead et al., 2022); hallucinations; aggression; and other behaviors considered to be inappropriate (Swierkosz-Lenart et al., 2019). Importantly, these behaviors are a reflection of a high level of distress felt by the person with dementia and are distressing to loved ones and those providing care (NICE, 2018). In fact, BPSD and caregiver burden have been found to co-exist in a bidirectional relationship, such that BPSD worsens caregiver burden while caregiver burden, in turn, worsens the severity and frequency of BPSD (Isik et al., 2019). This cyclical relationship results in negative impacts within the dyad, including poor quality of life and increased risk for morbidities (Isik et al., 2019).

The prevalence and manifestation of BPSD can vary widely based on both stage and type of dementia (i.e., early-onset AD, late-onset AD, frontotemporal dementia, Lewy body dementia, etc.) (Altomari et al., 2022; Laganà et al., 2022; Cognat et al., 2023), personality traits (Young et al., 2019), and even genetic underpinnings (Scassellati et al., 2020). Prevalence estimates range anywhere from 10% to 90% based these various factors (Altomari et al., 2022; Laganà et al., 2022; Pozzi et al., 2023). Furthermore, several psychosocial variables can influence BPSD, such as intrapersonal factors and circumstances (personality, physical pain, unmet needs), interpersonal interactions (caregiver stress and depression, and negative communication styles of caregivers), and environmental triggers (change in dwelling, unfamiliar surroundings, too much or too little stimuli) (Kales et al., 2015). The nature of this broad variation can be a source of discord among professionals regarding several diagnostic and treatment considerations. The debate surrounding terminology, best practices, and human rights issues has since permeated social medial platforms, advocacy groups, and international dementia organizations (Cunningham et al., 2019; Wolverson et al., 2019, 2021; Steele et al., 2020). This lack of consensus and even controversy exists at least in part due to the negative impacts on the physical and psychological health of persons with dementia as the result of the label of BPSD itself, as well as the way in which it is perceived, assessed, and medically treated (Cunningham et al., 2019).

This paper aims to discuss two of these potentially controversial considerations, (1) the assessment of BPSD, including the possible biases, misinterpretations, and stigma, and (2) the management of BPSD, including potential risks of pharmacological intervention which underscore the preference for non-pharmacological interventions, particularly from a lens of a person-centered framework. While BPSD can present in any form of dementia, this review will mainly focus on AD, as it is the most common form of dementia, and with the recognition that other forms of dementia present with neuropsychiatric symptoms as the most salient symptomology as opposed to memory impairment.

## Considering assessment

The complexity of BPSD are reflected in the current challenges of assessment and management. Despite several decades of research and enormous number of instrument iterations, a single satisfactory assessment tool has not been developed (although the Neuropsychiatric Inventory is likely the most widely accepted) (Pozzi et al., 2023). Moreover, caregiver report bias and subjectivity are major barriers to objective assessment in the surfeit of available tools, which carry considerable heterogeneity in the expertise required to administer (Pozzi et al., 2023). For comprehensive review of these tools, please see Pozzi et al. (2023). A further challenge of assessment is maintaining the objectivity with which to evaluate BPSD in order to accurately distinguish BPSD that are the result of neurodegeneration vs. BPSD that are the product of reasonable reactions to situational triggers. As language functions begin to deteriorate over the course of the disease, this delineation can become increasingly obscure and further adds to the challenge of objective interpretation of the subjective experience in persons with dementia. This can create considerable difficulty for both the person with dementia and caregivers, especially as the disease progresses, whereby persons with dementia may experience an *increase* in needs but a *decrease* in the ability to communicate or fulfill them independently (Ferreira et al., 2017). Unmet needs encompass a wide spectrum of basic necessities that range from physiological needs (i.e., toileting, bathing, hunger, thirst) to boredom. Consistent with the biopsychosocial model of dementia first developed by Cohen-Mansfield (2000) and later expanded by Spector and Orrell (2010), current manifestations of the disease are the net result of biological, psychological, and environmental factors; some of which may be relatively fixed or amenable to change (Gagliese et al., 2018). The biopsychosocial model of dementia includes manifestations of BPSD and can be applied to determine whether some manifestations are the result of unmet needs (e.g., pain, mood), indicating an opportunity for intervention.

The Camberwell Assessment of Need for the Elderly (CANE) is a common metric used to identify unmet needs in long-term care centers, and studies have shown that the lack of daytime activities and company are among the most commonly reported (Ferreira et al., 2016; Michelet et al., 2022). Of the current assessment tools available, CANE is standardized, validated, and widely used to assess the presence of unmet needs formal and informal care systems because of its integrated evaluation of wide range of needs (Carvacho et al., 2021; Mansfield et al., 2022). Importantly, there are several assessment tools [e.g., The Johns Hopkins Dementia Care Needs Assessment (JHDCNA)] for informal caregivers and community-residing persons with dementia to identify medical care, services, safety needs, and support that are often unmet (Black et al., 2013; Kipfer and Pihet, 2020). To evaluate the reliability, validity, and relevance of these assessments, Kipfer and Pihet (2020) conducted systematic review of available assessments for informal caregivers. Their final review evaluated 14 needs assessment instruments and found the Partnering for Better Health-Living with Chronic Illness: Dementia (PBH-LCI:D) to have the best psychometric evidence with the Questionnaire Consultation Expectations (EAC) also partially supported, while the remainder had limited or no support for psychometric soundness; the Scales Measuring the Impact of Dementia on Carers (SIDECAR) demonstrated promising results for content validity; and none of the instruments could be assessed for criterion validity due to lack of a gold-standard (Kipfer and Pihet, 2020). In many cases, a proxy is used to identify unmet needs, although there is growing recognition of people with dementia's ability to accurately convey their needs (Mansfield et al., 2022). Based on this premise, Mansfield et al. (2022) recently developed a self-report measure to identify unmet needs in community-residing persons with dementia, the Unmet Needs Instrument for Dementia (UNI-D) which demonstrated acceptable internal consistency, face and construct validity, and acceptability to persons with dementia in their study of 95 participants. Consisting of 26 items, the UNI-D surveys unmet needs across five domains, health and wellbeing; dementia support; activities and hobbies; daily tasks; and access to support, which may help identify meaningful support needed by persons with dementia (Mansfield et al., 2022).

Unfortunately, unmet needs are disturbingly common in persons with dementia and are associated with increased distress, depression, and anxiety; decreased quality of life; BPSD, premature institutionalization; and premature mortality (Ferreira et al., 2017). Importantly, unmet needs are inextricably associated with BPSD and quality of life (Ferreira et al., 2016; Michelet et al., 2022). Interestingly, the consequences of unmet needs demonstrate consistent overlap with the consequences of BPSD, which include significant distress in persons with dementia and caregivers; adverse outcomes; misuse of medications; long-term hospitalization (Laganà et al., 2022); reduced quality of life; early institutionalization (Kales et al., 2015; Altomari et al., 2022); and excess morbidity and mortality (Kales et al., 2015). As such, it is critical to rule out behavior changes that are the result of unmet needs before diagnosing a person with dementia with BPSD, as that label can carry negative consequences. To confound this matter further, persons with dementia may possess a lowered threshold to stress (Hall and Buckwalter, 1987; Felstead et al., 2022), yet are often living in highly stressful or uncertain situations. Considering the multidirectional relationships between chronic stress within the dyad, unmet needs, and BPSD, the importance of valuable assessment tools is critical for both the clinician and informal caregiver, especially considering the increased likelihood of institutionalization associated with BPSD. Early identification of unmet needs may point to the need for non-pharmacological and person-centered approaches to meeting such needs in the home setting to avoid, or at least delay, negative outcomes.

## Considering the person

Tom Kitwood, the social psychologist who pioneered the person-centered care approach to persons living with dementia (and from whose work this article title was inspired), brought some of these psychosocial influences to the forefront of research by raising the methodological issue of conceptualizing “the problem” of dementia as exclusive to individual, rather than the interpersonal milieu (Kitwood, 1993). This is still reflected in literature today as there is a disproportionate amount of research that targets the caregiver perspective, rather than the perspective of the person living with dementia, especially with regard to the experience of stigma (Lion et al., 2021). The negative perceptions that are born from this view of the “the problem” also give rise to what he termed, “malignant social psychology,” which are the unintentional but damaging behaviors of healthy (i.e., cognitively intact) others toward persons with dementia, such as outpacing, invalidation, infantilization, banishment, and stigmatization (Kitwood, 1993). A few examples of these interactions may include treating or speaking to someone patronizingly as if they are a child (infantilization); not allowing a person to use their preserved abilities or failing to assist in completion of initiated tasks (disempowerment); using deception to promote compliance (treachery); and failing to acknowledge one's subjective reality and feelings (invalidation) (Kitwood, 2019). Malignant social psychology is especially pertinent with respect to the label of BPSD, such that this label positions the person with dementia as one who is no longer a whole person, but rather they are “less than,” or “tainted,” which embodies the very definition of stigma (Goffman, 1974; Pachankis et al., 2018). Using a medical lens through which to view BPSD can increase the susceptibility to pathologize what would be otherwise rationale behaviors in certain settings, especially when considering one's individual predicament (Kitwood, 1993). This may be especially pertinent when a person with dementia is placed in a long-term care facility. In the words of Viktor Frankl, “An abnormal reaction to an abnormal situation is normal behavior.” Frankl et al. (2006, p. 20). When considering the perspective of the person with dementia, numerous constructs of their situation are “abnormal,” which can lead to reasonable reactions such as fear, sadness, anger, anxiety, and depression. Failing to consider the reasoning behind a person's behavior, whether situational or circumstances of unmet needs, is deeply discrediting and can create a cycle of increased stress, distress, and associated BPSD (Warren, 2022). This act of discrediting the subjective experience and behaviors of persons with dementia can have a profound impact on their quality of life. Indeed, BPSD and associated stigma attached to its label can lead to negative outcomes including a reduced sense of wellbeing, poor health outcomes, psychological distress, increased probability of institutionalization, and increased likelihood of unmet needs (Warren, 2022).

## Considering language

The stigma attached diagnostic labels has been evident across a myriad of medical conditions (e.g., mental illness, obesity, HIV, dementia) can be as limiting as the disease itself (Corrigan, 2014). Moreover, it may lead to psychological suffering, social isolation, delays in timely diagnosis, and barriers to necessary healthcare for many individuals (Bacsu et al., 2020; Rosin et al., 2020). Therefore, words via dialogue and medical labels can greatly influence the thoughts, beliefs, emotions, and behaviors of others toward persons with dementia (Wolverson et al., 2019). As such, it's important to consider the language we use to medically characterize persons with dementia and their potential downstream consequences.

The terminology used to characterize behavior changes in persons with dementia has been a topic of debate across disciplines, countries, and individuals (Cunningham et al., 2019; Wolverson et al., 2019). Discussions are ongoing to identify a psychosocial term(s) that reflects the complexity of human emotion and behavior from a more person-centered standpoint (Michelet et al., 2022). Wolverson et al. (2019) explored terminology preferences in 378 multidisciplinary professionals and stakeholders and found “unmet need” was most frequently ranked as first choice, followed by “behavior that challenges,” “BPSD,” and “stress and distress” as the most preferred among of a wide variety of responses. The qualitative portion of their survey reflected themes that emphasized the need for a dementia-friendly term that promotes personhood and avoids stigmatizing and objectifying a person's experience (Wolverson et al., 2019).

Wolverson et al. (2021) conducted another study to explore the opinions of persons with dementia about BPSD terminology. Of the 54 participants, 28.3% preferred the term “unmet need” to describe perceived changes in behavior. While a clear consensus of a preferred term was not found, responses reflected a concern among persons with dementia that the language used to characterize behavior changes would influence how they were viewed and treated, as well as their own self-perceptions (Wolverson et al., 2021). Burley et al. (2023) also investigated the opinions of persons with dementia and their families and caregivers about preferred terminology and what they found helpful or unhelpful responses to behavior changes. The interviews of 21 people with dementia and 20 care partners revealed that discriminatory treatment and the use of medicalized terminology was unhelpful, and the term “changed behaviors” was most preferred (Burley et al., 2023). They further showed that persons with dementia found helpful responses were those in which clear professional support pathways were given, as well as supportive environments that promote physical, cognitive, social, and spiritual engagement (Burley et al., 2023). Participants also conveyed desire for improvement in dementia-related stigma, societal education about dementia, and a focus on living well with dementia (Burley et al., 2023).

Psychoemotional reactions to the diagnosis of any type of dementia are difficult to effectively portray in words alone. The author, Terry Pratchett, upon learning of his diagnosis of AD relayed that he felt as if he had two diseases, “one was Alzheimer's and the other was knowing I had Alzheimer's” (Pratchett, 2015, p. 1). It is common for those diagnosed with AD, as well as their care partners, to experience a great amount of fear and angst in anticipation of what is perceived to be an inexorable and irreversible loss of self (Halewood, 2016; Bitenc, 2020). The psychological distress experienced by persons with dementia, their caregivers, as well as persons with mild cognitive impairment (MCI) who have not yet (and may never) developed dementia, cannot be overstated (Alzheimer's Disease International, 2012; Burgener and Buckwalter, 2018). In fact, the diagnosis of dementia is a key nodal point of disempowerment (Low et al., 2018), and by extension, the label of BPSD further exacerbates what is already a major threat to self and identity. Likewise, the ways in which healthy others change their behaviors toward persons with MCI or AD create a scenario of a classic confirmation bias, in which healthy others selectively pay attention to information that affirms their assumptions of impairment and in this case, can result in the negative associations and prejudices inherent in the diagnosis of dementia (Sabat, 2006). In this way, not only do persons with dementia experience dementia-related stigma, but those who are at higher risk for developing dementia (i.e., person with MCI or known genetic predispositions) may also be subject to dementia-related stigma (Stites et al., 2017).

The broad continuum of dementia types and AD subtypes feature significant symptom overlap but differing primary clinical features [i.e., explicit memory loss (AD), visuospatial deficit (posterior cortical atrophy), personality changes (Lewy Body Dementia, Frontotemporal dementia)]. Yet, neuropsychiatric symptoms can occur at any stage and in any type of dementia, even in possible pre-dementia stages such as MCI which may represent an early indication of dementia (Godin and Theou, 2020). When neuropsychiatric changes or BPSD-like symptoms emerge in preclinical stages, they are termed “Mild Behavioral Impairment” (MBI) (Godin and Theou, 2020) and typically represent a long-standing change in behavior (Kassam et al., 2022). Persons with MCI plus MBI have a great chance of developing dementia (Mallo et al., 2018). Herein lies another conundrum of discerning a diagnostic label from reasonable psychological states due to the current situation and begets the question of how and to what extent a diagnostic descriptor contributes to malignant social psychology and stigmatizing behaviors. Depending on the context and behavior of clinicians and healthy others, it could provide a comfort knowing the symptoms experienced and exhibited by persons with dementia are a product of brain degeneration, or, they could worsen the psychosocial milieu and associated outcomes of the person with dementia.

Family members, the public, caregivers, and even healthcare professionals are not immune to the negative perceptions associated with dementia-related stigma, as well as the psychiatric stigma associated with mental illness (Zhang et al., 2023). The stigma and stereotypes associated with dementia have led to a broad range of human rights issues, as persons with dementia are positioned and perceived as incapable of agency and therefore no longer bearers of human rights (Cahill, 2018; Steele et al., 2020). Yet, progressive loss of autonomy does not equate to loss of agency. However, as autonomy deteriorates, persons with dementia are often isolated and at greater risk for abuse and neglect, while third parties are legally empowered to authorize decisions they deem is in their best interest (Cahill, 2018; Steele et al., 2020). Human rights movements for persons with dementia are beginning to emerge to try to improve the lives of persons with dementia and legitimize their experience, but much work has yet to be done to eradicate the stigma associated with BPSD and dementia.

## Considering management

Ideally, proper evaluation and accurate assessment of behavior changes can identify both the origin of BPSD and appropriate care options to promote quality of life for persons with dementia and caregivers alike. Management generally incorporates a combination of pharmacological and non-pharmacological approaches, but psychotropic drugs are typically used off-label and carry a high rate of adverse events (Cognat et al., 2023). International guidelines set forth by the National Institute for Health and Care Excellence (NICE) recommend non-pharmacological approaches as the first line of treatment for BPSD, yet use of psychotropic drugs remains high (NICE, 2018; Eikelboom et al., 2023). Non-pharmacological interventions are generally variegated and have demonstrated well-established efficacy in reducing the frequency and severity of BPSD (Plante-Lepage et al., 2022; Tampi et al., 2022). Most non-pharmacological interventions (e.g., massage therapy, pet therapy, multisensory stimulation, reminiscence therapy, music therapy) do not have any adverse effects and require minimal to moderate investment (Scales et al., 2018). Yet, the use and implementation of non-pharmacological interventions remains insufficient (Eikelboom et al., 2023).

Person-centered non-pharmacological interventions have demonstrated superior benefit to pharmacological interventions to persons with dementia and caregivers (Bennett et al., 2021; Losada-Baltar and Jiménez-Gonzalo, 2021), and lack the adverse effect profile. A systematic review by Scales et al. (2018) identified three practice themes including “sensory practices (aromatherapy, massage, multi-sensory stimulation, bright light therapy), psychosocial practices (validation therapy, reminiscence therapy, music therapy, pet therapy, meaningful activities), and structured care protocols (bathing, mouth care)” (Scales et al., 2018, p. S88). Despite the evidence-base in clinical trials, translation into practice requires an individualized approach, as some interventions may or may not be enjoyable depending on how they are offered to the individual (e.g., music preference, comfort around animals) (Scales et al., 2018). Furthermore, implementation into practice necessitates training, education, reminders, and audits of professionals and strategies for best practices to do so are still lacking (Bennett et al., 2021). This consideration notwithstanding, a systematic review focusing on implementation strategies found that utilizing multiple strategies to increase use of non-pharmacological interventions that are incorporated into daily routines of clinicians have shown improvements in BPSD (Bennett et al., 2021).

The risks posed by psychotropic drugs include worsening cognitive and functional decline (Muniz et al., 2020; Cognat et al., 2023), increased risk of falls and cerebrovascular events (Muniz et al., 2020; Tampi et al., 2022), fractures (Abraha et al., 2017), and death (Muniz et al., 2020; Tampi et al., 2022; Cognat et al., 2023). What's more, many persons with dementia are already physically frail, which further elevates their risk of adverse events to psychotropics, and the available clinical trials investigating drug therapy and BPSD reflect that functionally impaired older persons with dementia have not been adequately considered (Seibert et al., 2021). When warranted for severe BPSD, they provide only modest symptom control in the majority of patients (Abraha et al., 2017). However, despite their low success rate and significant adverse effects, psychotropic medications are widely used non-specifically, and remain a pervasive issue ubiquitously (Muniz et al., 2020; Plante-Lepage et al., 2022; Eikelboom et al., 2023). To address inappropriate use of medications, the CHROME (CHemical Restraints avoidance Methodology) criteria were developed, and studies have found that implementation of these guidelines is easily adapted by physicians and institutions (Muniz et al., 2020). However, research has found that even though non-pharmacological approaches and caregiver training reduce BPSD in persons with dementia, many formal caregivers still rely on antipsychotics (Plante-Lepage et al., 2022).

## Recharacterizing BPSD

A successful recharacterization of BPSD through person-centered, human-rights perspectives must entail a reframing of the dementia narrative, the success of which necessitates improved education on several levels. Persons with dementia are often viewed principally by their incapabilities, rather than their preserved functions (Warren, 2021).

Knowledge gaps about the syndrome of dementia and associated BPSD persist as additional barriers to best practices. Therefore, efforts to improve quality of life and quality of care in persons with dementia must include appropriate educational interventions for formal and informal caregivers, as well as the public. Education must aim toward and understanding of the underlying causes of BPSD as well as non-pharmacological approaches, lest caregivers become quickly overwhelmed and resort to pharmacological approaches and institutionalization (Hinkle and Pontone, 2023). However, due to the heterogenous nature of BPSD, along with copathologies that are more the rule than the exception, it is difficult for providers to anticipate how BPSD will manifest (Hinkle and Pontone, 2023).

Physician perceptions of preparedness, capability, wiliness, and management approaches differ greatly (Eikelboom et al., 2020), which easily translates to ill-prepared caregivers, for whom education and support is still lacking (Braun et al., 2019). Furthermore, family caregivers sometimes feel their level of physical and emotional distress associated with BPSD is not evident until a crisis point is reached (Braun et al., 2019). Studies examining barriers to the management of BPSD from the caregivers perspective include lack or absent education and services; lack of respite care; delayed recognition of BPSD; poor communication and unrealistic expectations (Braun et al., 2019). Helpful approaches included educational support; day centers; reminiscence therapy and music interventions (Braun et al., 2019); maintaining personhood; responding positively to BPSD; explanation and bargaining; controlling upsetting thoughts; and getting respite care (Leung et al., 2021). Furthermore, recent studies have demonstrated that positive aspects of caregiving are reinforced by knowledge of BPSD, which promotes friendly caregiving attitudes, and high self-efficacy (Hu et al., 2022). Knowledge of BPSD better prepares the caregiver to manage BPSD and maintain perceptions of self-efficacy in this regard.

Recently, dementia awareness initiatives have been emerging globally to promote helping behaviors, facilitate social inclusion, and reduce stigma directed toward persons with dementia (Matsumoto et al., 2022). Improving dementia literacy and dementia friendly environments may maximize time in the home and encourage inclusion in daily life (Matsumoto et al., 2022). Enhancing knowledge has also been associated with improvements of public and caregiver attitudes toward persons with dementia (Shin and Lee, 2019; Cowan, 2021).

## Discussion

This brief review aimed to explore some of the controversies and disparities experienced by persons with dementia and caregivers. Stigma is a pervasive issue across many pathologies and cultures. As such, it is incumbent upon providers as those who oversee the assessment, education, and management of BPSD to mediate the challenges embedded in the diagnostic assessment and labels used today. If beauty is in the eye of the beholder, as the old adage goes, then perhaps at times, pathology is likewise a product of perception and bias. If a person with dementia becomes depressed because family members rarely visit or their spouse views/treats them differently, is that BPSD or an adjustment disorder that would otherwise be reasonable in persons without the label of dementia? The position of this paper is such that BPSD is a major problem, which is *often but not always* the result of neurodegeneration. Some symptoms are certainly the unfortunate cause of neuronal deterioration and functional network aberrations. However, even in severe symptoms such as hallucinations, it is incumbent on the clinician to rule out other causes (e.g., adverse drug reactions, drug interactions, malnutrition, and other medical disorders) before assigning origin to BPSD. That being said, the clinical feature of BPSD is undisputed. BPSD causes immense burden and distress to all parties, but the ways in which it is labeled and characterized could profoundly affect the outcomes for persons with dementia and their caregivers. Perhaps, reframing behavioral and psychological symptoms of dementia (BPSD) to something more akin to adjustment symptoms of dementia may be helpful. Assigning a more person-centered label such as “adjustment” unless otherwise specified (i.e., adjustment depression, adjustment anxiety, adjustment apathy), may facilitate caregivers to search for understanding of symptoms due to other causes than solely dementia. This may further implication and provocation to caregivers to uncover to what they are adjusting (i.e., unmet needs, loneliness, a new living situation surrounded by strangers; chronic pain, etc.), as opposed to symptoms naturally inherent in the disease process. The goal is to find a term which can detach the automatic assumption of neuropsychological expressions from the diagnosis of dementia *just enough* to consider other causes through a lens of personhood rather than pathology. Something more reflective of unmet needs is enticing, such as behavioral and psychological symptoms of unmet needs, but that would rule out symptoms of true neuronal deterioration inherent in the disease process. Perhaps, vagueness of the former is a friend in this regard.

A person-centered approach that prioritizes human rights must entail a reciprocal approach, between the clinician, person with dementia, and caregiver. Furthermore, prioritizing the voice of patients and family members can help inform management strategies based on individual preferences and personality characteristics in this regard. Stakeholders and the lay public must also be informed in a greater effort to dispel stigma and optimize relationships and quality of life for the immense, and rapidly growing population who are experiencing, or will experience cognitive decline.

## Author contributions

AW: Writing—original draft.
